# Future climate impacts on maize farming and food security in Malawi

**DOI:** 10.1038/srep36241

**Published:** 2016-11-08

**Authors:** Tilele Stevens, Kaveh Madani

**Affiliations:** 1 Centre for Environmental Policy, Imperial College London, London SW7 2AZ, United Kingdom

## Abstract

Agriculture is the mainstay of Malawi’s economy and maize is the most important crop for food security. As a Least Developed Country (LDC), adverse effects of climate change (CC) on agriculture in Malawi are expected to be significant. We examined the impacts of CC on maize production and food security in Malawi’s dominant cereal producing region, Lilongwe District. We used five Global Circulation Models (GCMs) to make future (2011 to 2100) rainfall and temperature projections and simulated maize yields under these projections. Our future rainfall projections did not reveal a strong increasing or decreasing trend, but temperatures are expected to increase. Our crop modelling results, for the short-term future, suggest that maize farming might benefit from CC. However, faster crop growth could worsen Malawi’s soil fertility problem. Increasing temperature could drive lower maize yields in the medium to long-term future. Consequently, up to 12% of the population in Lilongwe District might be vulnerable to food insecurity by the end of the century. Measures to increase soil fertility and moisture must be developed to build resilience into Malawi’s agriculture sector.

Agriculture is the primary source of income and provides the main livelihood for the world’s poorest people in developing countries^1^,^2^,^3^. The nature of climate change (CC) impacts is variable^4^ and will drive varying responses in crops and regions around the world^5^. CC is expected to have positive and negative impacts on crop yields^1^,^6^, that will have important consequences for food prices and food security^7^. The risks to adverse effects of CC are expected to be greatest in Least Developed Countries (LDCs), particularly in sub-Saharan Africa (SSA) and South East Asia^8^ with the most vulnerable communities^3^,^9^,^10^. Projections from the 2013 Intergovernmental Panel on Climate Change (IPCC) report indicate that in Southern Africa (SAF), the most profound effects of CC will be temperature increases over 4 °C by the end of the century and less rainfall^11^. The agriculture sector in SSA is highly vulnerable to CC because of the high dependence on rainfed agriculture, which makes the region susceptible to adverse weather events such as flooding and drought^12^,^13^. This is exacerbated by the region’s immense reliance on natural resources, high population growth rates, acute poverty levels, and poor infrastructure^3^,^8^,^9^,^10^.

Located in the eastern part of SAF (Fig. 1), Malawi is an LDC^14^ with a huge poverty challenge^13^. The agriculture sector plays a pivotal role in Malawi’s socioeconomic wellbeing and poverty alleviation. It contributes to as much as 27% of the nation’s gross domestic product (GDP)^15^ and employs 85% of the population^16^. Over 90% of the agriculture sector consists of smallholder farmers^17^. Rainfed agriculture is widespread in Malawi, with less than 5% of famers using irrigation^13^. There is some evidence to suggest that Malawi’s fluctuating historical trend in GDP growth is a consequence of changes in seasonal rainfall (Supplementary Fig. S1); this emphasises the vulnerability of the country to CC. Over 95% of farmers in Malawi cultivate to meet their subsistence needs, highlighting the importance of farming for the nation’s food security^18^.

Maize (*zea mays* L) is the nation’s staple and most important food crop^19^. Maize is by far the most widely grown crop in the country and accounts for as much as 80% of the cultivated land^20^. It is thought that the country’s food security is defined by maize harvests and access to maize^21^,^22^. Up to 59% of Malawi’s land is used for cultivation^23^. In addition to maize, other crops that are important for food security in Malawi are cassava, groundnuts, peas, potatoes, pulses, and sorghum^19^,^20^. In Supplementary Table S1, we present a summary of recent (2013) production and international trade value information about these crops in comparison to maize.

High population growth in Malawi is a key driver behind the increasing national maize food requirement over the last few decades (Fig. 2). Fluctuating trends in Malawi’s GDP closely follow those of agricultural productivity and maize production (Fig. 3). These agricultural and economic fluctuations are reflected in food prices. Malawi’s domestic maize prices are more volatile than international prices^18^,^19^ which can have devastating social outcomes during food shortages when prices rise sharply^24^.

In recent years Malawi has experienced droughts and floods, which emphasise the country’s susceptibility to impacts of CC^25^. In 2015 poor maize harvests, 30% lower than in the previous harvest season, were recorded^26^. In a recent report, the Malawi Vulnerability Assessment Committee (MVAC)^26^ attributed the poor harvests to the late onset of rains in the 2014–2015 maize growing season, followed by continuous heavy rain in early 2015 which caused widespread flooding and affected 25 of the country’s 28 districts. Consequently it was projected that up to 2.8 million people would require humanitarian assistance in the period between October 2015 and March 2016; an intervention which equates to US$ 33 million^26^. Similarly, in 2005, the country experienced the worst maize harvests in a decade because of a lack of rain during the crucial maize growth stages^27^. As a result the yield was only 0.76 tonnes per hectare (t/ha), 40% below the long term average^27^. This equated to 57% of the national food requirement and left 5 million people requiring food aid^27^. To mitigate these impacts, in the mid-2000s, the Government of Malawi (GoM) implemented a Farm Input Subsidy Program (FISP) which achieved ground-breaking success in tackling food security^27^. Nonetheless, the GoM campaigns for the introduction of alternative approaches that can build resilience into the agriculture sector to mitigate the impacts of CC^28^.

Given the high vulnerability of Malawi to CC, it is important to understand how CC impacts can affect food security and economic growth. Several studies have reported the impacts of CC on maize farming in Malawi, with conflicting results as to whether the effects would be positive or negative^13^,^20^,^29^,^30^. In this study, we focused on Lilongwe District (Fig. 1), located in central Malawi at an altitude of 1149 metres^31^. Lilongwe District is Malawi’s dominant cereal producing region^32^ with a humid sub-tropical climate^33^. There are two distinct seasons in Malawi, the rainy season from November to April, and the dry season from May to October. Lilongwe District’s predominant soil type is sandy clay loam^34^. Malawi is densely populated, with a population of 17.2 million that is steadily increasing at 3% per annum^15^,^35^. In 2010, the reported population for Lilongwe District was 2.1 million^36^.

## Results

### Regional climate trends for rainfall and temperature

Large scale changes in monthly rainfall and maximum temperature, projected by five Global Circulation Models (GCMs) under CC scenario Representative Concentration Pathways (RCPs) 4.5 and 8.5, for three future time periods (2020s, 2050s and 2080s) relative to a baseline period (1971 to 2000) are illustrated graphically (Figs 4 and 5). The interquartile ranges of the box and whisker plots (Figs 4 and 5) reflect the inherent uncertainty in the output from the GCMs. Our results suggest relatively high uncertainty around future rainfall patterns, with more rainfall in some months and less rainfall in others, but no strong increasing or decreasing trend for annual rainfall (Fig. 4). Temperature is expected to increase in the future, with higher temperature projected for RCP8.5 compared to RCP4.5 (Fig. 5). The greatest increases in temperature are projected for the 2080s. The results show greater uncertainty for the projected rainfall changes compared to the projected temperature changes.

We applied a three step statistical approach to address this uncertainty by: (i) calculating the relative ability of each GCM to simulate both rainfall and temperature for each month, (ii) generating discrete and continuous probability distribution functions (PDFs) of CC scenarios for each month, and (iii) converting the PDFs to cumulative distribution functions (CDFs) using the Beta probability distribution (Supplementary Figs S2 and S3). We performed a sensitivity analysis of the GCM outputs by stochastically modelling the projected rainfall and temperature and reporting them at different probability percentiles (25%, 50% and 75%). Supplementary Tables S2, S3 and S4 list the values of the sum squared of error (SSE) and Beta distribution function parameters which we used to develop the continuous PDFs and CDFs. The relatively low values of SSE indicate the suitability of the Beta distribution to produce time series for both temperature and rainfall^37^. In some cases we obtained relatively high values for SSE (underlined in Supplementary Tables S2, S3 and S4), however we believe that this does not greatly affect the results.

When compared to the baseline period, future rainfall projections (Fig. 6) suggest that different outcomes can be expected for monthly rainfall changes at different probability percentiles. Based on the future rainfall projections (Fig. 6), with the exception of RCP4.5 in the 2020s where more rainfall is expected throughout the dry season, we expect rainfall to increase at the beginning of the dry season (May) and decrease thereafter (June to October). In the 2020s there could be more rainfall at the start of the rainy season (December) but less rainfall thereafter (January to April). In the 2050s, we can expect more rainfall during some months (December to March) of the rainy season under RCP4.5 and relatively high rainfall variability under RCP8.5. At the end of the century, in the 2080s, we project the rainy season to be shorter for both RCP4.5 and RCP8.5 because of less rainfall at both the beginning and end of the season. The results we obtained for temperature projections predict higher temperature in the future (Fig. 7). However, the maximum and minimum expected level of temperature change could be variable depending on the CC scenario and future time period. For example, in the 2020s under RCP4.5, the greatest temperature increase is projected for November and the smallest change for May. Contrastingly, in the 2080s under RCP8.5, the maximum temperature increase is predicted for November and the minimum change for January.

### Downscaled climate trends for rainfall and temperature

We used the Long Ashton Weather Generator (LARS-WG5)^38^, a widely used stochastic weather generator (WG), to downscale the GCM outputs after successfully calibrating and validating for Lilongwe District. We applied statistical tests, results of which are presented in Supplementary Table S5, to assess the satisfactory performance of the LARS-WG5 before applying it to downscale the GCM outputs.

Historically, annual rainfall variability in Malawi is high which can be problematic when identifying long term rainfall trends^39^. Compared to the baseline period of 1971 to 2000, rainfall is expected to change by −7.3% to −0.4%, −2.0% to 11.0%, and −12.3% to −1.9% in the 2020s, 2050s and 2080s, respectively. Although the annual rainfall results did not reveal a strong increasing or decreasing trend, we expect more rainfall variability in the future. This is expected to be more pronounced in the medium to long-term future for RCP8.5 compared to RCP4.5. Compared to moderate changes in annual rainfall, we project reductions in rainfall in the rainy season for the future except for RCP4.5 in the 2050s where there could be more rainfall. These rainy season changes are expected to range from −8.6% to −2.8% in the 2020s, −3.0% to 7.5% in the 2050s, and −13.1% to −3.7% in the 2080s, compared to the baseline period.

We project future maximum temperatures to be in the range of 0.7 °C to 0.8 °C, 1.6 °C to 2.3 °C, and 2.1 °C to 3.3 °C in the 2020s, 2050s and 2080s, respectively. Consistent with the IPCC (2013) temperature projections, RCP8.5 results show a greater temperature increase compared to the RCP4.5 ones. This is because of more rapid socioeconomic and population growth, with limited CC mitigation and adaptation, under the RCP8.5 CC scenario. The results are also comparable with other findings for Lilongwe District: 0.5 °C to 0.9 °C, 1.3 °C to 2.2 °C, and 1.8 °C to 3.6 °C in the 2020s, 2050s and 2090s, respectively^30^; 1.8 °C by the 2050s^40^; and 1.8 °C to 2.2 °C by the 2050s^32^.

### Climate change impacts on maize yields

We assessed the effects of CC on maize yields, representing biophysical impacts, using the Food and Agriculture Organization (FAO) crop model, AquaCrop^41^. Prior to undertaking any crop modelling, we applied statistical tests to assess the ability of AquaCrop to reproduce maize yields. Our model validation results indicated that in general the AquaCrop model overestimated maize yields, as observed by other studies carried out in Malawi^42^,^43^ that used the same model. Thus, despite this limitation, the model was deemed to be fit-for-purpose.

Our findings from the maize yield modelling suggest that both increases and decreases in maize production are to be expected under CC. Compared to the baseline period (1971 to 2000), maize production could benefit from CC with yields ranging from 4.6% to 5.4% in the 2020s. This could be driven by increasing atmospheric carbon dioxide (CO_2_) concentrations coupled with the slight increase in temperature which would promote faster crop growth. The aforementioned moderate reduction in the amount of rainfall is not anticipated to pose significant adverse impacts on maize production in the short-term future. It is understood that under well watered conditions maize responds positively to increasing CO_2_ levels, up to a point, because of an increase in canopy size which translates to higher productivity and yields^44^ through CO_2_ fertilisation^6^. These findings for the 2020s contradict earlier CC impact studies in Lilongwe District which reported declines in maize yields in the short-term future^30^,^45^.

In the 2050s, maize production is expected to show little change under CC, with yield changes in the range of −1.2% to 1.0%. The CO_2_ fertilisation effect could still have a positive effect on maize growth in the mid-century. Under increasing temperatures, greater levels of evapotranspiration and a reduction in soil moisture levels can be expected to adversely affect maize growth^13^. These results are comparable to other studies in Lilongwe District which reported that in the 2050s, maize yields would be reduced by a moderate 5%^32^,^40^. However our findings contradict earlier studies which reported both significant increases in maize production ranging from 5% to 25%^13^, and notable decreases ranging from −14% to −7%^30^.

Maize production is expected to generally decrease, in the 2080s, with projected yield changes in the range of −3.0% to 0.2%. Increasing temperature is likely to have the biggest adverse effect on maize yields at the end of the century. Higher temperatures will shorten the maize growing season, cause capture of less solar radiation and result in decreased crop productivity of maize plants^40^. Furthermore, the low soil water content will reduce the amount of nitrogen that is taken up by maize plants^40^, which could significantly reduce maize yields^46^. In the long-term future, rainfall variability and a shorter maize growing season are likely to be key drivers for reduced maize yields^13^,^40^,^45^. The projected maize yield declines are moderate compared to a recent study by Msowoya^30^ who reported reductions in the range of −33% to −13% for the end of the century in Lilongwe District.

### Climate change impacts on food security

We evaluated the vulnerability of Lilongwe District to food security using national per capita maize requirement, as defined by MVAC and Lilongwe District population growth estimates based on the national United Nations (UN) population growth variants.

**Figure 2:**
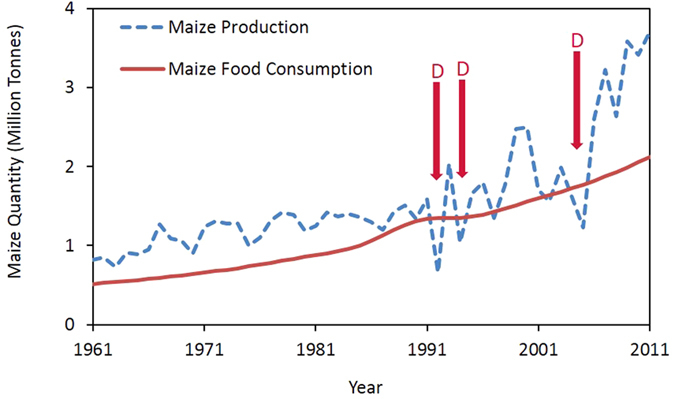
Malawi’s historical national maize production^67^ and maize food consumption. Due to high population growth^35^, and the prevalence of high maize consumption in the Malawian diet (approximately 382g/capita/day^52^), national maize requirement has increased steadily over the last few years. ‘D’ indicates notable drought years).

Due to the high population growth rate in Malawi and historical national maize requirement trends (Fig. 2), the amount of maize required to maintain food security in Lilongwe District is expected to steadily increase in the future. Based on the modelled future maize yields, we estimate that Lilongwe District could produce 0.5 million tonnes annually in the 2020s, 2050s and 2080s under the projected impacts of CC at different risk levels (probability percentiles). Under conditions of stable maize production, maize requirements could readily be met in the short to medium-term future (Fig. 8). Furthermore, with no adverse impacts on maize production, there could be maize production surpluses under both RCP4.5 and RCP8.5. Relative to 2013 FAO maize export values^47^ we estimate that the production surpluses could be worth between US$ 25 and 92 thousand annually on international maize markets.

In the 2080s, the projected lower maize yields, coupled with increasing population, could lead to maize shortages and translate to between 0.1% and 12.2% of the population in Lilongwe District being vulnerable to food insecurity. A recent study by the Overseas Development Institute (ODI)^48^ in Malawi, communicated that CC is expected to adversely affect all four facets of food security namely availability, access, utilisation and stability. Taking into account 2013 values reported by the FAO for maize imports^47^, we estimated that the deficit in maize production could equate to as much as US$ 355 thousand being invested annually in international markets to feed Lilongwe District’s growing population.

## Discussion

### Impacts of climate change on Malawi’s agricultural system and food security

Compared to other reported findings of massive reductions in maize yields in SSA and Malawi^10^,^30^,^45^,^49^, our study findings anticipate the decline in future maize yields in Lilongwe District to be moderate. This is because the projected changes for both the amount of rainfall and temperature increase, in the study area, are modest.

In the short-term future, farmers could benefit from CC due to the expected increase in maize yields under favourable climate conditions. It has been reported that despite the devastating projected impacts of CC, some areas in SSA could fare relatively well^1^. This could have important research and policy implications for Malawi’s agricultural sector.

Interestingly, higher maize production under favourable CC conditions could intensify the existing problem of poor soil fertility in Malawi. Accelerated crop growth can lead to high nutrient requirement and result in soil nitrogen deficiency^6^. We, therefore, envision that in the short-term future, the increasing demand for food and the positive impacts of CC on maize growth could adversely impact soil fertility. Given the moderate increases in temperature and relatively low decline in rainfall amount and variability, in the short-term future, impacts of soil fertility on maize yields far outweigh any predicted impacts of CC.

In the medium to long-term future increasing maize demand caused by a growing population, coupled with decreasing maize production, could exacerbate vulnerability to food insecurity. Lower maize production is expected to be driven by (i) higher rainfall variability leading to more droughts and floods, (ii) increasing temperatures, (iii) a higher frequency of years when farmers fail to sow crops due to late onset of rainfall^40^, and (iv) a shorter maize growing season^45^. By the end of the century, maize requirements are projected to be greater than production and would therefore lead to food shortages (Fig. 8) and higher maize prices.

### Policy implications for addressing projected impacts of climate change

In responding to the impacts of CC, the United Nations Framework Convention on CC (UNFCCC) advocates for the response of LDCs to be adaptation rather than mitigation. The two main schools of thought in CC adaptation strategy planning are sustainable resource management and research and development (R&D)^12^. Sustainable resource management includes consideration of soil, water, and crops; whilst under R&D, approaches such as the use of drought resistant and heat tolerant maize varieties could be considered^50^. Scholars hold the view that through its National Adaptation Programme of Action (NAPA), Malawi has the potential and opportunity to manage the threats and impacts of CC^13^. Through good field management practices such as irrigation and improved soil fertility, maize yields in developing countries can be increased from a range of 1 to 2 t/ha to as much as 11 to 14 t/ha^46^.

The GoM and a number of non-governmental organisations (NGOs), academics and authors recognise the important role that irrigation has to play in improving food security in Malawi^51^. With the Ministry of Agriculture and Food Security (MoAFS) reporting that only 14% of Malawi’s irrigation potential is currently being utilised^40^, there is a great opportunity to improve maize yields through adopting this approach.

Many farmers in Africa use crop diversification to build resilience into the agriculture sector^49^. The feasibility of such an approach is questionable especially in a nation such as Malawi where the prevalence of maize in the diet is very high^19^. Therefore crop diversification could raise big cultural questions, that is forsaking a traditional staple food - maize - for an alternative CC tolerant one^9^. Drought tolerant crops that have been suggested for Malawi include cassava, sorghum and millet^13^,^20^. Crop diversification has additional benefits for food security; it reduces malnutrition and places households in good stead to overcome volatile food prices^52^.

Given the projected impacts of CC on soil fertility in the short-term future, a priority for the GoM would be to improve soil fertility. It is a widely held view that by improving affordability of farm inputs including fertiliser, through Malawi’s FISP, there have been tangible benefits for food security^50^.

Soil composting^22^ and conservation agriculture (CA), through rotation of maize and cowpea crops^45^, have been recommended for Malawi. The application and effectiveness of CA methods has been exemplified in a study where maize yields in a region of Malawi were increased by 40%^42^. In their effort to promote the year 2015 as International Year of Soils, the FAO produced a series of radio programmes including one titled Chuma Chiri M’nthaka (“wealth is in the soil”) which was broadcasted in Malawi to promote integrated soil fertility management practices amongst smallholder farmers^53^.

In the possible absence of adverse impacts of CC, in the short-term future as demonstrated in this study, maize yields could be greatly improved. Better maize yields could translate to increased food security in Malawi where most of the maize farming is for subsistence use.

### Prospects for improved income

Being the main cereal-producing district in Malawi, and with the projected short-term positive impacts of CC, Lilongwe District could produce food surpluses. If these could reach the international food markets, under the expected global price increase for cereals^9^,^10^,^12^, the Malawian economy stands to possibly gain from the short-term impacts of CC. There are other reports of Malawi’s agriculture sector benefitting from CC; for example, a study in Mzimba District in the northern region of Malawi concluded that in the period between 2040 and 2070, 56% of farmers would gain from CC because of higher maize yields^29^. However, despite the projected attractive global prices and increasing global demand for maize, the ability of Malawi’s agriculture sector to become an established exporter is questionable given the need to feed a growing population.

**Figure 3:**
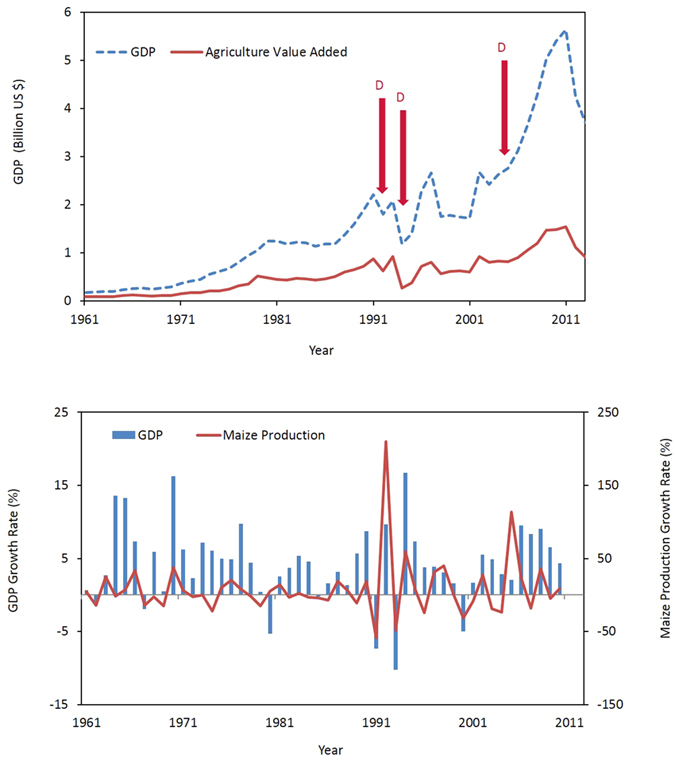
The significance of agriculture and maize production in Malawi’s economy shown by (**a**) Malawi’s agricultural added-value and total GDP (2015 US$ price time base)^15^ where ‘D’ indicates notable drought years, and (**b**) Malawi’s maize production^67^ and GDP growth^15^ over the last few decades.

**Figure 4:**
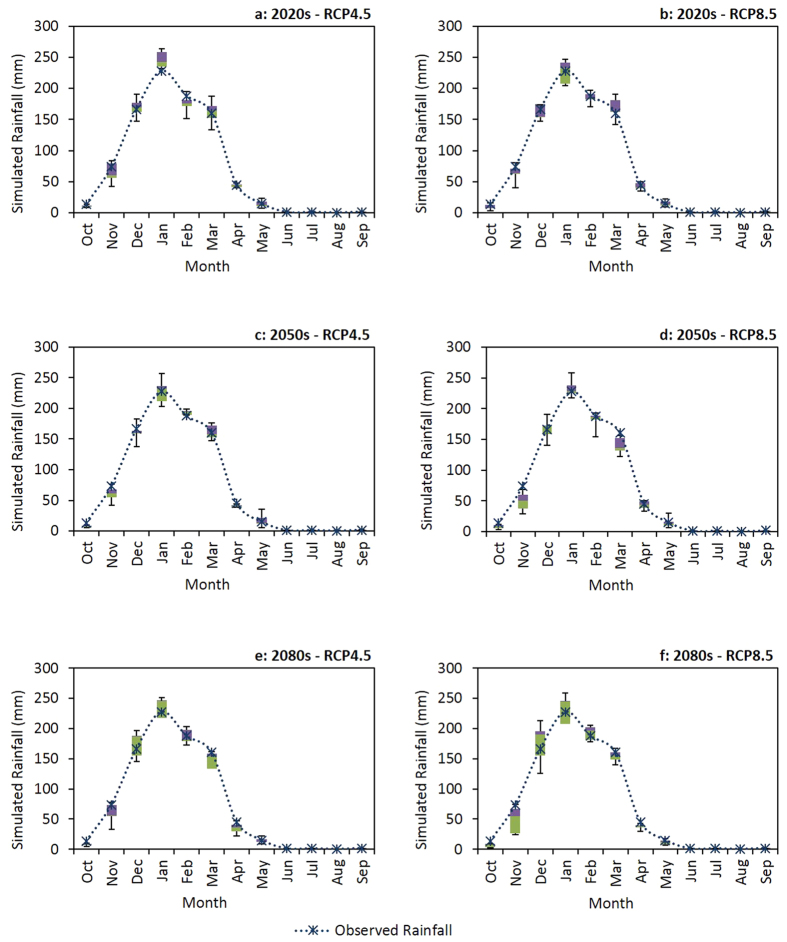
Large scale rainfall patterns projected by the GCMs, compared to the observed weather data (dotted line) from the baseline period (1971 to 2000). The box plot whiskers illustrate mean ± standard deviation, the boxes show the upper and lower quartiles, and the line in the middle indicates the median.

**Figure 5:**
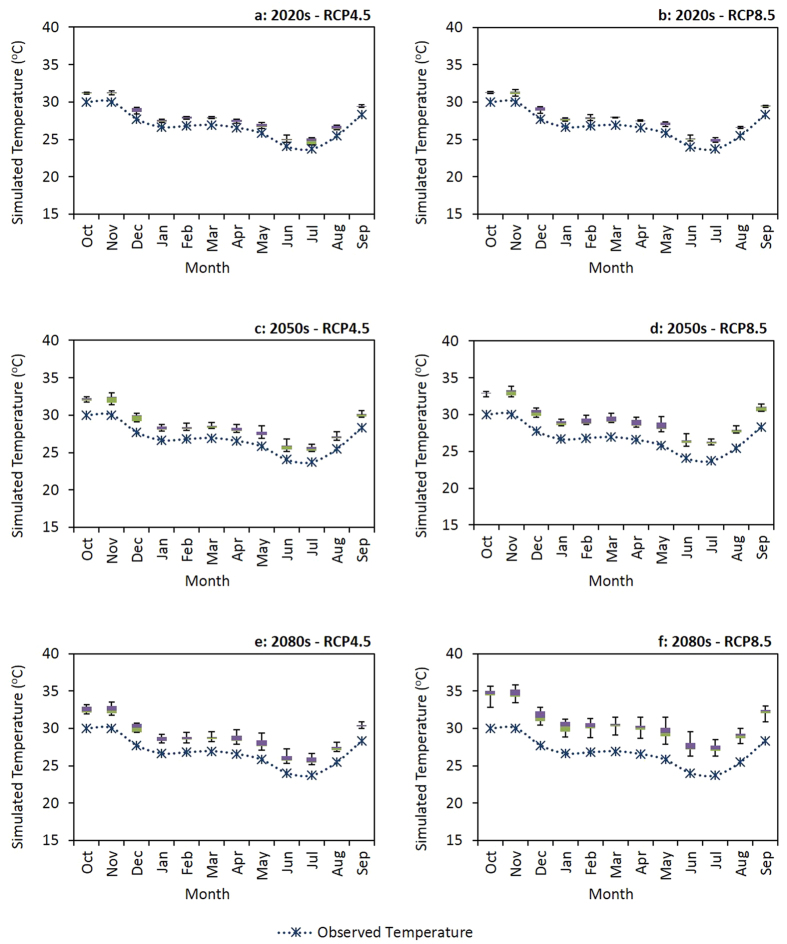
Large scale maximum temperature patterns projected by the GCMs, compared to the observed weather data (dotted line) from the baseline period (1971 to 2000). The box plot whiskers illustrate mean±standard deviation, the boxes show the upper and lower quartiles, and the line in the middle indicates the median.

**Figure 6:**
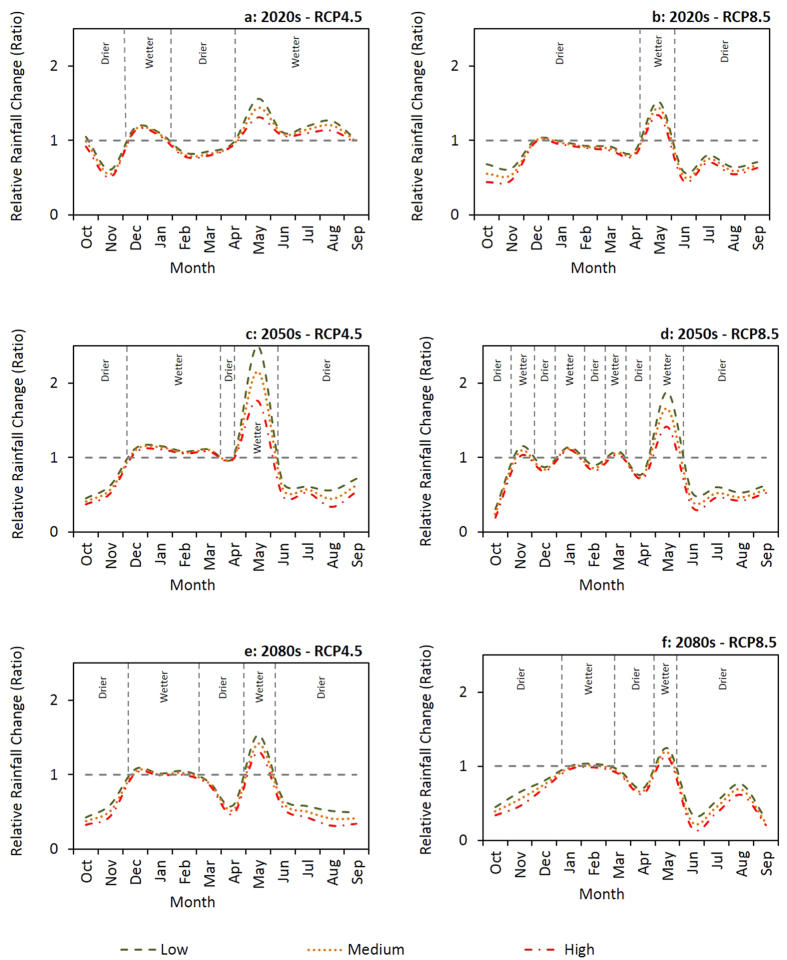
Estimated large scale rainfall changes, compared to the baseline period (1971 to 2000), at different CC risk level scenarios. The dotted line indicates no rainfall change. The rainfall change ratio is the ratio of the projected to baseline rainfall in each month.

**Figure 7:**
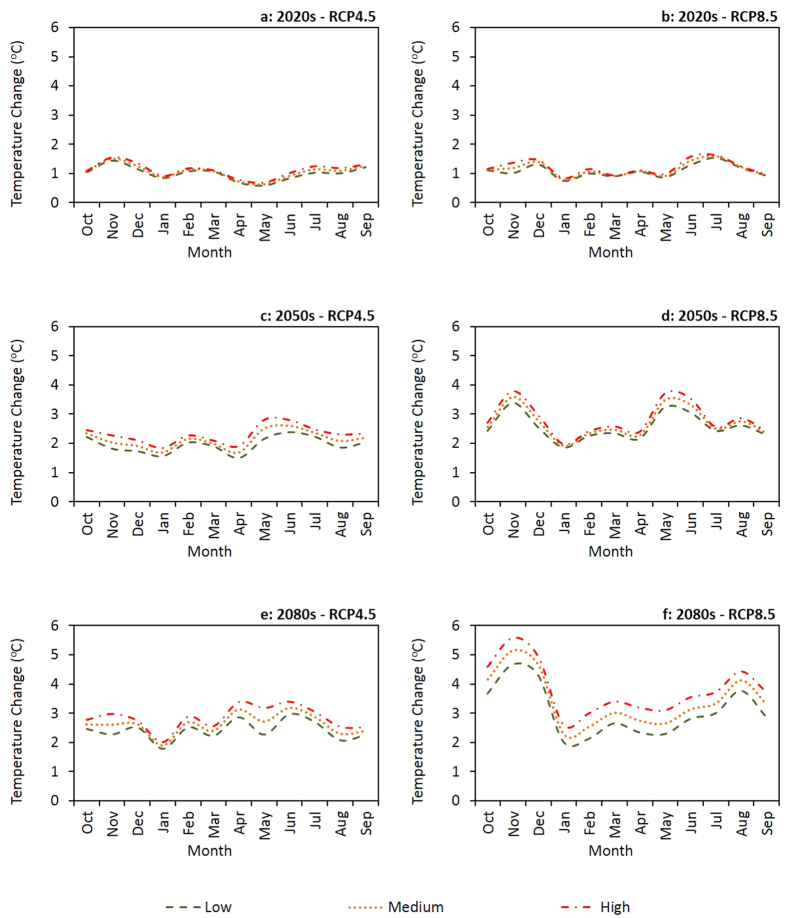
Estimated large scale temperature changes, compared to the baseline period (1971 to 2000), at different CC risk level scenarios for each month. We predict higher temperatures in the future under all the modelled CC scenarios.

**Figure 8:**
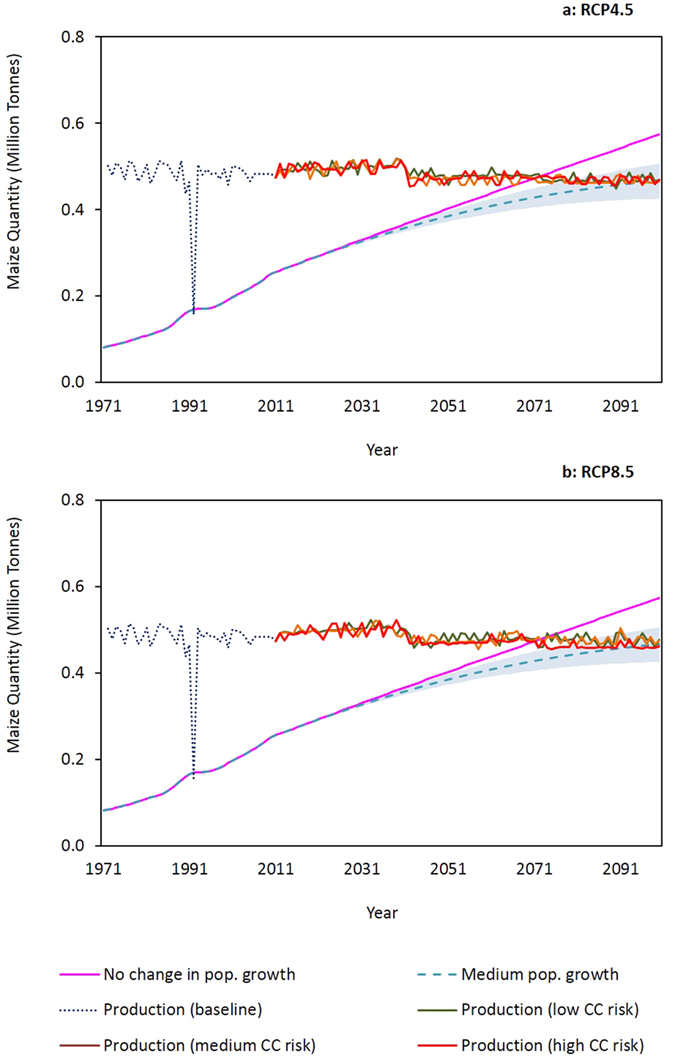
Estimates of maize production (simulated by AquaCrop) and maize requirements under different population growth projections in Lilongwe District under (**a**) RCP4.5 and (**b**) RCP8.5. The shaded area represents the upper and lower bounds of population growth calculated from UN population estimates^35^ and the reported population for Lilongwe District^36^. These graphs highlight low maize production during the 1991–1992 maize growing season, which was a drought year. Although not captured in the AquaCrop simulations, 1993–1994 and 2004–2005 were notable drought years in Malawi.

A major hindrance to the implementation of CC adaptation measures is poverty^2^. As mentioned previously, poverty increases the vulnerability of Malawi to CC impacts. A recurring challenge for the GoM is in mobilizing resources to respond to immediate food security crises whilst concurrently investing for the nation’s long-term future in agriculture^18^. What is more, the sustainability of agriculture as a source of income for Malawian households in the future has been questioned^20^. However, we believe that by investing in R&D and increasing agricultural productivity and yields, smallholder farmers could produce crop surpluses which could be sold to bring in extra household income. Such economic gains could increase per capita income, drive poverty levels down and empower smallholder farmers. Furthermore, it has been demonstrated empirically that higher income can produce tangible benefits for nutrition in Malawi^52^. Economic empowerment could allow smallholder farmers, who make up 90% of Malawi’s agriculture sector, to lift themselves out of poverty by increasing their access to education, health, and financial services^28^. By empowering Malawi’s poorest people, there could be tangible benefits for the whole nation. Given the pivotal role that the agriculture sector holds in the Malawian economy (Fig. 3a), any CC adaptation measures that can mitigate the projected adverse CC impacts are likely to benefit a significant number of farmers and the population.

### Study limitations

It is important to bear in mind the possible bias in the results from this study. Although we used state-of-the-art GCMs to predict future CC in Lilongwe District, climate projections are subjective. The acknowledged sources of uncertainty in GCMs are: uncertainty in future anthropogenic greenhouse gas (GHG) emissions and natural forcings; limited knowledge of current climate conditions; challenges of representing variability in future projections; and imperfections in the GCMs^54^. Moreover, with a small number of GCMs, caution must be applied, in interpreting our study results. A major drawback of using a small number of GCMs is that the CC projections could fail to account for seasonal and regional biases of climate model simulations, which could be overcome by using more GCMs. Additionally, we did not make adjustments for how our CC projections would affect distributions of dry and wet spells in the future. A more in-depth assessment of rainfall variability and temperature changes would highlight probable future duration of wet and dry spells. This could be particularly useful in Malawi where it is a widely held view that socioeconomic wellbeing is linked with seasonal rainfall (Supplementary Fig. S1). Other sources of bias in our results could come from having limited information about the farming conditions in Lilongwe District for the AquaCrop model calibration, and using a restricted set of assumptions for the food security vulnerability assessment. Furthermore, we did not consider other environmental parameters which could have an effect and influence the results presented here. Finally, we did not take into account pre-existing socioeconomic factors such as access to local and international markets; market conditions under the predicted increasing global demand for maize; changes in consumer preference; and potential interventions such as subsidies.

## Conclusion

We undertook a study to evaluate the impacts of CC on maize production and food security in Malawi’s Lilongwe District in three future time periods (2020s, 2050s and 2080s) compared to a baseline period (1971 to 2000), under RCP4.5 and RCP8.5 CC emission scenarios.

We downscaled the outputs from five GCMs using a commonly used stochastic WG, the LARS-WG5. We then used a three-step statistical approach to account for the uncertainty of GCM outputs. Through this process, a range of probability percentiles of projected rainfall and temperature changes were developed (25th, 50th, and 75th). We used the FAO crop model, AquaCrop, to simulate maize yields in the future under CC.

Our climate modelling results suggest that maximum temperature could increase by 0.7 °C to 0.8 °C, 1.6 °C to 2.3 °C, and 2.1 °C to 3.3 °C in the 2020s, 2050s and 2080s, respectively. Although the annual rainfall results did not reveal a strong increasing or decreasing trend, we expect more rainfall variability in the future. Under CC, maize yields are expected to both increase and decrease in the future. We predict that maize yield gains and losses would range from 4.6% to 5.4%, −1.2% to 1.0% and −3.0% to 0.2% in the 2020s, 2050s and 2080s correspondingly. The results reflect the opposing apparent effects of CC on crop production. Despite the projected high population growth rates for Malawi, maize requirements could be met in the short to medium-term future. In the 2080s, under the assumptions used in the study, lower maize production could result in between 0.1% and 12.2% of the population being at risk of food insecurity. We estimate that as much as US$ 355 thousand would need to be allocated annually for purchasing maize from international markets to feed the growing population of Lilongwe District alone.

Our findings highlight the importance and implications of future policy formulation and additional research with regards to CC adaptation measures. A reasonable path to achieve food security, under inevitable CC, could be through a holistic approach that considers sustainable resource management as well as economic and market factors. To meet the probable growing soil nutrient deficiency, caused by faster growth crop under CC in the short-term future, the GoM would need to consider measures that improve soil quality and fertility. To mitigate against the projected adverse effects of CC on maize yields and food security in the medium to long-term future, we consider irrigation and access to local and international markets to be key factors. Given the pivotal role that agriculture holds in Malawi’s economy and the imminent growing population, such practices are likely to have benefits that will drive income and social empowerment for many smallholder farmers in Lilongwe District and Malawi.

## Methods

### Climate change modelling

We used the outputs from five GCMs (Supplementary Table S6), under two emission scenarios (RCP4.5 and RCP8.5), described in the Fifth Assessment Report (AR5) of the IPCC, to make projections for rainfall and temperature in 2011 to 2040 (2020s), 2041 to 2070 (2050s) and 2071 to 2100 (2080s). For each GCM, we extracted daily time series of rainfall, temperature and short wave radiation for RCP4.5 and RCP8.5 for 2011 to 2100 as well as for the baseline period (1971 to 2000). Founded on the Coupled Model Intercomparison Project Phase 5 (CMIP5) multi-model experiment of the World Climate Research Programme, the RCPs are the 2013 CC emission scenarios published by the IPCC. The RCP4.5 scenario is characterised by CC policy and increased environmental sustainability resulting in stable GHG^11^,^55^. On the other hand the RCP8.5 scenario assumes high GHG emissions caused by high population growth and energy usage, low rates of development in developing countries and a heavy reliance on fossil fuels^11^,^55^. We compared the short (2020s), medium (2050s) and long-term (2080s) future changes in climate to the baseline period. We obtained historical observed weather data, collected by Chitedze Research Station in Lilongwe District, from the Department of Climate Change and Meteorological Services (DCCMS) in the Ministry of Natural Resources, Energy and Environment in Malawi.

Understanding performance of GCMs is an important aspect of CC science^54^. Therefore we assessed the uncertainty in the GCMs by applying a probability analysis method with bounded distribution functions in a three step approach^5^. In the first step, we assessed the ability of each GCM to reproduce baseline rainfall and temperature using the Mean Observed Temperature-Precipitation (MOTP) method^5^. We weighted each model by calculating, for both rainfall and temperature, the difference between the monthly mean observed values and the simulated values for the baseline period as follows^5^,^37^:


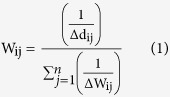


where W_ij_ is the weight of GCM j in month i, Δd_ij_ is the absolute difference in rainfall or temperature between the monthly mean simulated by GCM j in month i of the baseline period and the corresponding observed value, n equals five (the number of GCMs). It is worth highlighting some factors which we identify as limitations in the research methods in this study. Although our methodology tries to address the uncertainty of the GCMs, using a limited number of GCMs could introduce error into the climate modelling procedure^5^.

In the subsequent step, we generated discrete PDFs showing the relationship between the difference in monthly rainfall and temperature and the calculated weight of the corresponding GCM (Supplementary Figs S2a and S3a). This involved calculating the ratio of rainfall and absolute difference for temperature in each future time period, under each RCP, and comparing it to the baseline data for each month as follows^5^:


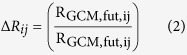



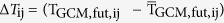


where ΔR_ij_ and ΔT_ij_ are CC scenarios of rainfall and temperature in month i and year j, R_GCM, fut, ij_ and T_GCM, fut, ij_ are the rainfall and temperature for each GCM in month i and year j in the future, R_GCM, base, i_ and T_GCM, base, i_ are the average simulated rainfall and temperature for each GCM in month i for the baseline period. At each future time period, at each emission scenario, we plotted 24 PDFs (12 for temperature and 12 for rainfall for each month); therefore we developed a total of 144 PDFs for the three future time periods under RCP4.5 and RCP8.5. To make CC projections about the future, we developed time series of rainfall and temperature from continuous PDFs. We fitted the Beta distribution function (equation (4)) onto each discrete PDF to convert it to a continuous PDF (Supplementary Figs S2b and S3b)^37^:


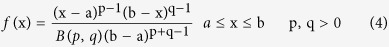


where x is rainfall or temperature, p and q are the shape parameters for the Beta distribution function, a and b are the minimum and maximum rainfall or temperature changes, and B(p,q) is the Beta function given by equation (5) and equation (6).


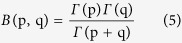



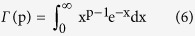


We used the maximum likelihood estimation method to identify the shape parameters (p and q) for the Beta distribution function. We calculated these by minimising the SSE as follows^5^,^37^:


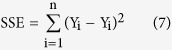


where y_i_ is the calculated weight for each GCM, Y_i_ is the estimation of the Beta function, and n equals five (the number of GCMs).

In the final step, we converted the PDFs to CDFs so that future rainfall and temperature changes could be calculated at different probability percentiles (Supplementary Figs S2c and S3c). In our risk assessment interpretation we considered that (i) a low CC risk level scenario represents low temperature (25% probability percentile) and high rainfall (75% probability percentile)^5^,^30^, (ii) a high CC risk level scenario represents high temperature (75% probability percentile) and low rainfall (25% probability percentile)^5^,^30^, and (iii) the 50% probability percentile represents a medium risk level scenario of CC^37^.

We used the LARS-WG5 stochastic WG^38^ to downscale the GCM outputs in three distinct steps. In the model calibration step, we analysed the observed baseline (1971 to 2000) data (rainfall, maximum and minimum temperature, and hours of sunshine) to determine their statistical characteristics. We simulated weather data at the same grid point (−14.13 South and 33.38 East) as was used for the extraction of GCM data. To validate the LARS-WG5 model, we assessed its performance by comparing frequency distributions, mean values and standard deviations of the observed and simulated data of the baseline period using statistical tests (Kolmogorov-Smirnov test, T-test and F-test)^56^. We used these statistical tests to check the significance and the reliability of LARS-WG5 to predict future climate data, with reference to the baseline period, at the 0.01 significance level. Once we had deemed the LARS-WG5 performance to be acceptable, we used it to simulate future weather data. To manage the uncertainty of natural variability in the LARS-WG^5^, we generated ten 30-year time series for each future time period at the different probability percentiles. We used the average of these ten time series for the subsequent crop modelling step.

### Maize yield modelling

We used the FAO crop model, AquaCrop^41^, to simulate future maize yields under CC in three distinct steps: (i) calibration, (ii) validation, and (iii) simulation of future maize yields. A full list of parameters used to calibrate the model are summarised in Supplementary Table S7. In all three steps, evapotranspiration data (ET_o_) for the evaporation of water from the maize crop surfaces was required. We calculated this from weather data (maximum and minimum temperature, mean relative humidity, wind speed and hours of sunshine) using the ET_o_ calculator which uses the FAO Penman-Monteith method described by Allen *et al*.^57^. We used a number of sources to obtain data for the model calibration to the farming conditions and practices in Lilongwe District including literature^34^,^40^,^43^,^58^,^59^,^60^,^61^ and a consultation with a research scientist at Chitedze Agricultural Research Station. Based on the maize yield reported by the MoAFS in the year 2000, we calibrated the model. We validated the model using maize yields from the years 2001 to 2005 and five statistical indicators (Pearson coefficient of determination, relative root mean square error, normalized root mean square error, Nash-Sutcliffe and Willmott’s index of agreement)^41^. We found the model’s performance satisfactory and used it to make projections for future maize yields using baseline period weather data, the outputs from the LARS-WG for future weather data, and future CO_2_ concentrations^62^. We used the Pearson coefficient of determination to independently assess the relationships between maize yields and rainfall, temperature and CO_2_ concentrations.

The crop model calibrated in this study can be improved further by obtaining more user-specific information^63^ through controlled field experiments to mimic the farming practices in Lilongwe District. Additionally, we did not consider the effects of pests and diseases, and response of different maize cultivars. With respect to growing seasons, we only accounted for the rainy season maize cultivation and did not consider the widespread cultivation of maize in the dry season in Malawi.

### Food security modelling

We modelled impending food security by assessing maize deficits and surpluses from estimates of maize production and maize requirement. We calculated maize production as follows:


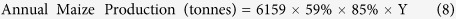


where 6159 square kilometres (Km^2^) is the area of Lilongwe District^31^, 59% is the proportion of cultivated land in Malawi^23^, 80% is the proportion of cultivated land that is used for maize farming in Lilongwe District^20^ and Y is the simulated maize yield (in t/ha) from AquaCrop for that year.

We calculated maize requirement as follows:


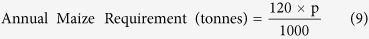


where 120 Kilograms (Kgs) is the MVAC estimate for annual maize requirement per person^64^, p is the population estimate for that year based on UN population growth estimates^35^ and the reported population for Lilongwe District^36^. By using the three different UN future population estimates for Malawi, we included a sensitivity analysis in the future projections of food security vulnerability in Lilongwe District.

We calculated maize deficit and surplus as the difference between maize requirement and maize production:


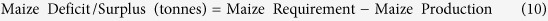


We calculated the proportion of the population in Lilongwe District that could be vulnerable to food insecurity in any given year as follows:


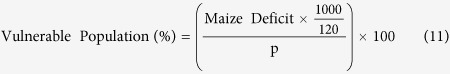


where p is the population estimate for that year based on UN estimates^35^.

Our food security vulnerability assessment can be improved in future studies by including other factors in the Household Economy Approach (HEA) framework, such as effects of maize yields on GDP and food price^65^.

### Estimating financial value of projected maize production and requirements

We estimated the monetary (import and export) value of the projected maize surpluses and deficits using values reported by the FAO (2013)^47^ for the 2013 international trade value of maize in Malawi. We did this as follows:


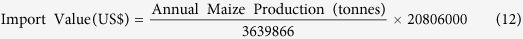


where US$ 20,806,000 was the import value of 3,639,866 tonnes of maize^47^.


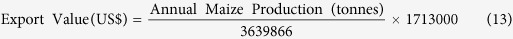


where US$ 1,713,000 was the export value of 3,639,866 tonnes of maize^47^.

## Additional Information

**How to cite this article**: Stevens, T. and Madani, K. Future climate impacts on maize farming and food security in Malawi. *Sci. Rep*. **6**, 36241; doi: 10.1038/srep36241 (2016).

## Supplementary Material

Supplementary Information

## Figures and Tables

**Figure 1:**
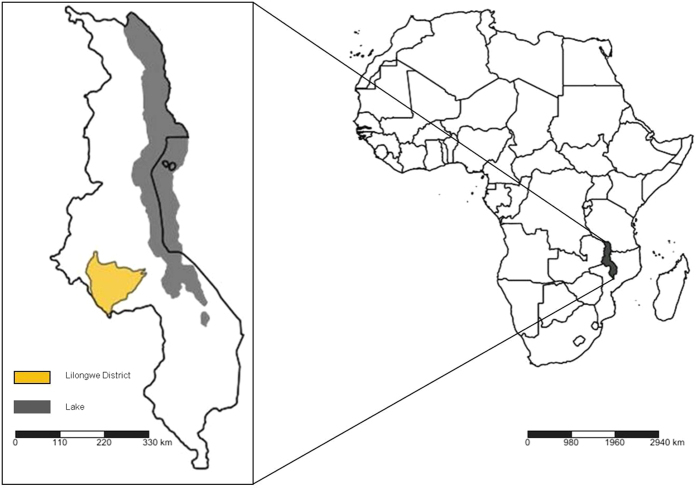
Map of Malawi and location of Lilongwe District. The maps were created using an online tool, SimpleMappr^66^.
