# Supplementary material for: Future climate impacts on maize farming and food security in Malawi
Source: Sci Rep. 2016 Nov 8;6:36241. doi: 10.1038/srep36241 (PMC5099946; doi:10.1038/srep36241)
Supplement: Supplementary Information [file srep36241-s1.pdf]

Future climate impacts on maize farming and food security in Malawi

Tilele Stevens and Kaveh Madani

Centre for Environmental Policy, Imperial College London, London SW7 2AZ, United Kingdom

Correspondence and requests for materials should be addressed to T.S.

(tilele.stevens14@alumni.imperial.ac.uk) and K.M. (k.madani@imperial.ac.uk)

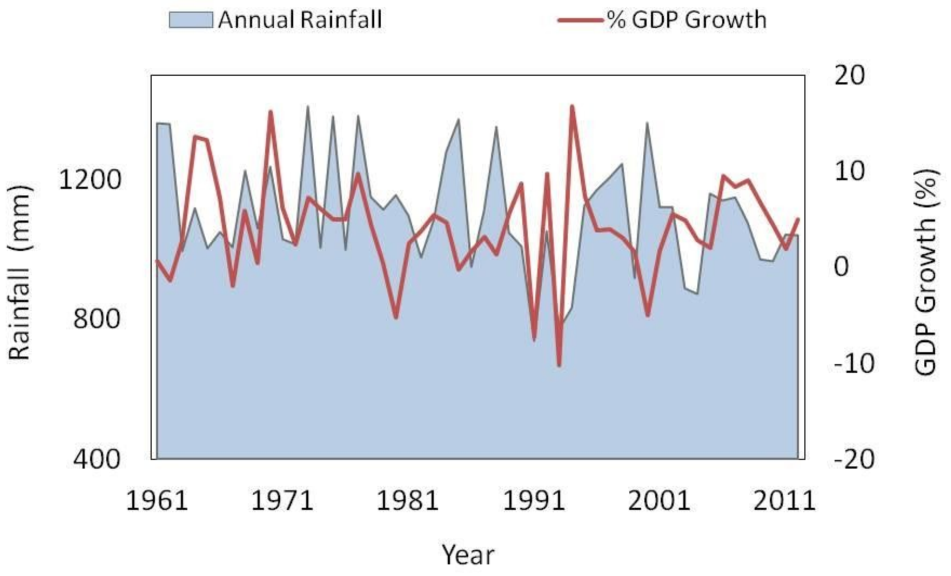

Supplementary Figure S1. Rainfall variability and economic growth[4] in Malawi over the last few decades. The graph shows similar trends in the country's Gross Domestic Product (GDP) growth and annual rainfall. This emphasises the vulnerability of the country's agriculture sector to climate change, which could cause changes in rainfall. Rainfall data were obtained from the Department of Climate Change and Meteorological Services (DCCMS) in the Ministry of Natural Resources, Energy and Environment in Malawi.

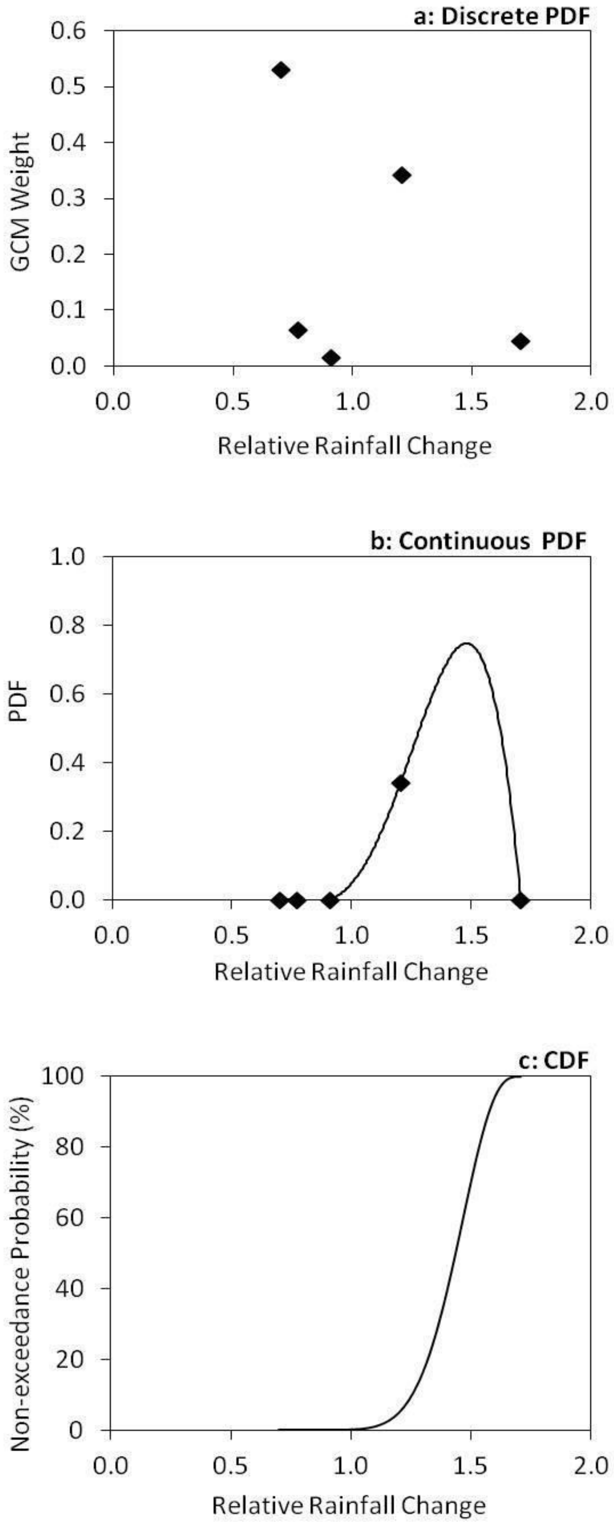

Supplementary Figure S2. Example of output graphs from statistical probability assessment used to address uncertainty in GCM outputs. This is an example of (a) discrete probability distribution function (PDF), (b) continuous probability distribution function (PDF), and (c) cumulative distribution function (CDF) for projected rainfall changes for May in 2020s under RCP8.5.

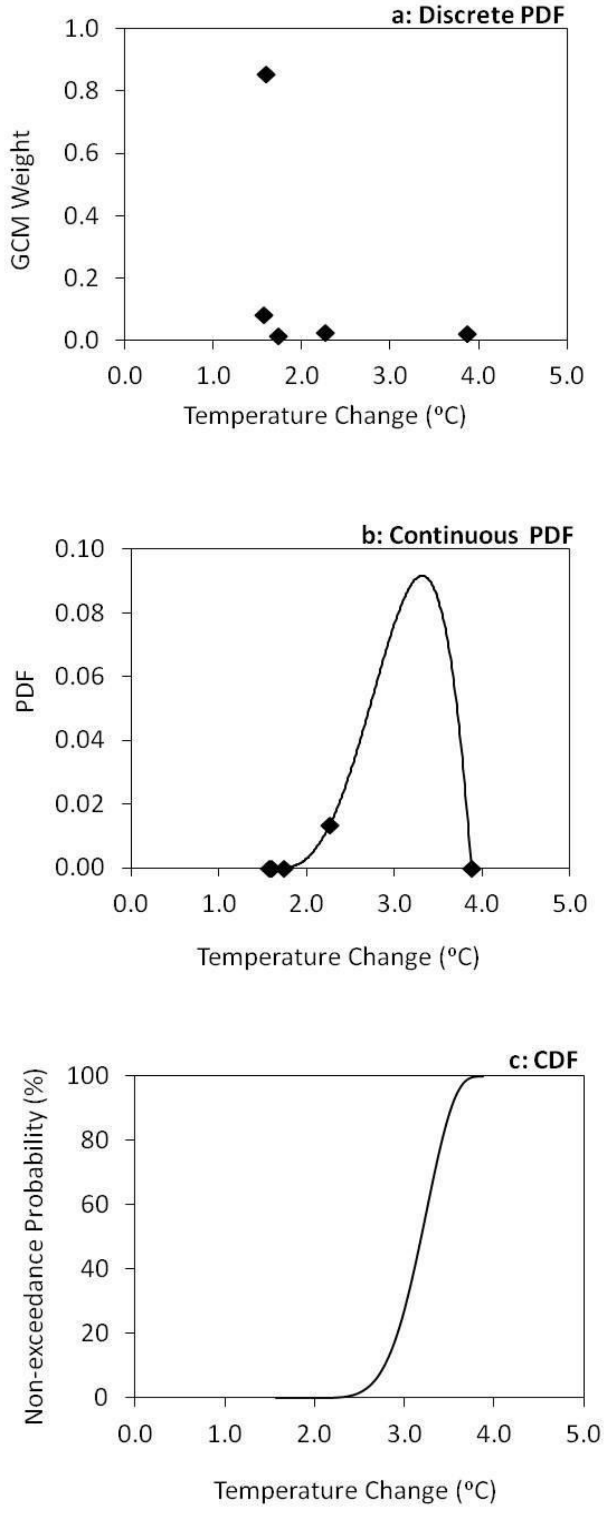

Supplementary Figure S3. Example of output graphs from statistical probability assessment used to address uncertainty in GCM outputs. This is an example of (a) discrete probability distribution function (PDF), (b) continuous probability distribution function (PDF), and (c) cumulative distribution function (CDF) for projected temperature changes for November in 2080s under RCP4.5.

Supplementary Table S1. Production and trade value of crops significant for food security in Malawi (2013 data)[47]

Crop	Area Harvested (hectares)	Production (tonnes)	Yield (tonnes/hectare)	Import Value (US$)	Export Value (US$)
Cassava	211,089	4,813,699	22.8	n/a	n/a
Groundnuts	362,824	380,800	1.0	n/a	58,806,000
Maize	1,676,758	3,639,866	2.2	20,806,000	1,713,000
Peas	53,000	40,000	0.8	915,000	17,247,000
Pulses	769,558	621,613	0.8	1,538,000	28,463,000
Potato	258,585	4,535,955	17.5	233,000	35,000
Sorghum	89,399	86,242	1.0	2,000	189,000

Supplementary Table S2. Estimated values for Beta distribution function parameters for rainfall and temperature changes for the 2020s. The relatively low values of the sum squared of error (SSE) values indicate the suitability of the Beta distribution to produce time series for both temperature and rainfall[37]. In some cases relatively high values were obtained for SSE (underlined in tables).

Rainfall

	RCP4.5					RCP8.5				
**Month**	**SSE**	**p**	**q**	**a (min)**	**b (max)**	**SSE**	**p**	**q**	**a (min)**	**b (max)**
**Jan**	<u>12.12</u>	3.00	5.00	1.02	1.19	<u>1.34</u>	6.22	7.78	0.87	1.08
**Feb**	0.29	3.00	10.00	0.74	1.05	0.27	3.00	11.00	0.87	1.06
**Mar**	0.42	2.76	14.00	0.75	1.21	0.42	3.91	15.09	0.81	1.21
**Apr**	0.33	2.85	8.15	0.91	1.10	0.28	3.00	14.00	0.75	1.18
**May**	0.30	7.94	5.06	0.62	1.95	0.29	9.00	3.51	0.70	1.71
**Jun**	0.35	16.14	2.75	0.77	1.14	0.34	3.75	11.00	0.33	1.08
**Jul**	0.41	9.00	2.52	0.69	1.27	0.41	2.51	3.49	0.61	0.95
**Aug**	0.28	9.00	2.93	0.62	1.38	0.29	2.00	8.00	0.48	1.05
**Sep**	0.38	15.00	2.57	0.69	1.03	0.39	2.80	15.20	0.57	1.20
**Oct**	0.17	4.69	2.31	0.62	1.16	0.41	2.88	7.12	0.21	1.45
**Nov**	0.22	2.00	10.20	0.44	1.15	0.23	2.00	5.12	0.35	1.06
**Dec**	0.27	17.00	2.50	0.84	1.20	0.17	9.29	4.08	0.87	1.05

Temperature

	RCP4.5					RCP8.5				
**Month**	**SSE**	**p**	**q**	**a (min)**	**b (max)**	**SSE**	**p**	**q**	**a (min)**	**b (max)**
**Jan**	0.15	12.08	7.92	0.57	1.07	0.09	2.56	9.00	0.66	1.27
**Feb**	0.07	5.00	2.97	0.82	1.30	0.29	5.00	11.00	0.77	1.75
**Mar**	<u>0.74</u>	16.00	3.00	0.77	1.14	0.12	2.99	8.00	0.86	1.07
**Apr**	<u>0.63</u>	2.94	10.06	0.58	1.26	0.34	9.49	3.00	0.84	1.15
**May**	0.27	2.18	16.00	0.52	1.51	0.27	2.62	13.00	0.77	1.68
**Jun**	0.37	3.97	13.03	0.63	1.90	<u>0.72</u>	3.40	2.60	0.83	1.88
**Jul**	0.09	5.03	2.97	0.51	1.50	0.41	12.94	2.00	0.89	1.68
**Aug**	<u>0.89</u>	3.00	4.00	0.78	1.52	0.17	8.98	10.02	1.06	1.40
**Sep**	0.18	7.00	3.00	0.88	1.43	0.16	2.15	16.00	0.90	1.26
**Oct**	0.10	2.35	12.00	1.00	1.41	0.26	2.00	12.03	1.08	1.47
**Nov**	0.09	10.64	3.00	0.90	1.66	0.48	2.94	3.06	0.55	1.85
**Dec**	0.26	6.00	3.00	0.59	1.57	0.24	11.86	4.14	0.62	1.65

Supplementary Table S3. Estimated values for Beta distribution function parameters for rainfall and temperature changes for the 2050s. The relatively low values of the sum squared of error (SSE) values indicate the suitability of the Beta distribution to produce time series for both temperature and rainfall[37]. In some cases relatively high values were obtained for SSE (underlined in tables).

Rainfall

	RCP4.5					RCP8.5				
**Month**	**SSE**	**p**	**q**	**a (min)**	**b (max)**	**SSE**	**p**	**q**	**a (min)**	**b (max)**
**Jan**	0.25	12.36	2.64	0.88	1.19	0.23	7.23	2.77	0.98	1.17
**Feb**	0.16	11.00	2.99	1.01	1.08	0.10	3.00	3.77	0.73	1.02
**Mar**	0.15	17.06	2.94	0.91	1.13	0.42	13.00	2.85	0.76	1.12
**Apr**	0.30	15.00	2.66	0.85	1.02	0.37	4.92	13.08	0.63	1.11
**May**	0.29	3.05	2.03	0.64	3.10	0.29	3.32	2.00	0.60	2.25
**Jun**	0.35	2.80	9.00	0.33	1.40	0.35	2.72	6.28	0.16	1.08
**Jul**	0.41	3.18	10.00	0.46	0.94	0.41	3.30	14.11	0.32	1.33
**Aug**	0.24	2.90	7.00	0.13	1.23	0.29	2.61	16.00	0.35	1.25
**Sep**	0.37	6.00	8.00	0.24	1.15	0.08	2.43	6.57	0.43	0.96
**Oct**	0.36	2.77	10.23	0.31	0.80	0.16	2.49	12.00	0.10	0.96
**Nov**	0.22	2.47	9.00	0.44	1.08	0.29	11.00	2.70	0.38	1.24
**Dec**	0.25	12.00	2.55	0.77	1.16	0.28	2.83	11.00	0.76	1.14

Temperature

	RCP4.5					RCP8.5				
**Month**	**SSE**	**p**	**q**	**a (min)**	**b (max)**	**SSE**	**p**	**q**	**a (min)**	**b (max)**
**Jan**	<u>0.51</u>	3.00	4.00	1.26	2.28	0.11	4.00	13.00	1.72	2.58
**Feb**	0.48	7.00	2.49	1.25	2.45	0.46	7.57	8.43	1.90	2.82
**Mar**	0.27	8.36	4.64	1.34	2.35	0.20	3.08	2.72	2.00	2.86
**Apr**	0.35	2.15	2.85	1.14	2.46	0.30	3.51	7.49	1.88	3.15
**May**	0.30	3.00	2.00	1.17	3.34	0.36	5.62	2.38	1.96	4.07
**Jun**	<u>0.74</u>	7.18	3.82	1.25	3.30	<u>0.74</u>	6.28	2.70	1.64	3.98
**Jul**	0.09	6.00	2.00	1.44	2.64	0.41	8.99	15.00	2.15	3.06
**Aug**	0.05	2.47	2.00	1.32	2.69	0.11	4.27	2.00	2.03	3.05
**Sep**	0.15	3.70	2.00	1.49	2.55	0.16	5.10	8.00	2.07	2.78
**Oct**	0.16	5.27	2.73	1.69	2.67	0.08	2.32	2.68	2.13	3.07
**Nov**	0.08	3.48	5.38	1.27	3.26	0.10	7.00	5.70	2.45	4.48
**Dec**	0.01	2.91	3.04	1.28	2.58	0.03	3.46	2.54	1.85	3.41

Supplementary Table S4. Estimated values for Beta distribution function parameters for rainfall and temperature changes for the 2080s. The relatively low values of the sum squared of error (SSE) values indicate the suitability of the Beta distribution to produce time series for both temperature and rainfall[37]. In some cases relatively high values were obtained for SSE (underlined in tables).

Rainfall

	RCP4.5					RCP8.5				
**Month**	**SSE**	**p**	**q**	**a (min)**	**b (max)**	**SSE**	**p**	**q**	**a (min)**	**b (max)**
**Jan**	0.13	5.00	8.00	0.94	1.09	0.15	4.06	10.15	0.90	1.16
**Feb**	13.69	3.27	2.73	0.91	1.11	15.32	2.55	2.45	0.92	1.09
**Mar**	0.01	3.11	5.85	0.82	1.01	14.21	2.89	4.11	0.84	1.06
**Apr**	0.32	2.99	10.01	0.36	1.11	0.30	3.00	7.00	0.55	0.93
**May**	0.29	6.50	4.00	0.73	1.83	0.40	15.00	3.84	0.44	1.36
**Jun**	0.34	5.54	6.46	0.33	0.96	0.36	2.00	7.00	0.04	1.07
**Jul**	0.41	4.00	6.23	0.22	0.94	0.35	3.12	4.88	0.20	0.85
**Aug**	0.29	2.94	8.00	0.13	1.21	0.30	9.87	5.13	0.15	0.96
**Sep**	0.37	2.55	9.45	0.22	1.18	0.37	2.96	16.00	0.15	0.78
**Oct**	0.18	2.53	5.47	0.24	0.68	0.11	6.00	7.00	0.14	0.69
**Nov**	0.26	3.87	7.13	0.26	1.00	0.31	2.97	5.03	0.26	1.07
**Dec**	0.24	10.39	4.61	0.80	1.15	0.28	3.89	12.00	0.60	1.25

Temperature

	RCP4.5					RCP8.5				
**Month**	**SSE**	**p**	**q**	**a (min)**	**b (max)**	**SSE**	**p**	**q**	**a (min)**	**b (max)**
**Jan**	0.40	2.75	10.25	1.63	2.93	0.32	2.00	6.42	1.63	4.61
**Feb**	0.48	9.00	2.82	1.25	3.11	0.45	2.76	4.24	1.25	4.62
**Mar**	0.24	6.60	5.00	1.48	3.08	0.22	4.59	5.41	1.48	4.86
**Apr**	0.34	6.00	2.98	1.49	3.90	0.07	3.00	6.23	1.49	5.49
**May**	0.30	2.38	2.62	1.34	4.25	0.04	2.00	8.00	1.77	6.66
**Jun**	0.74	8.94	4.00	1.58	3.87	0.34	2.28	8.00	2.26	6.55
**Jul**	0.41	7.00	2.90	1.64	3.36	0.11	2.35	4.55	2.39	5.32
**Aug**	0.27	2.80	3.20	1.56	3.17	0.12	4.77	2.00	2.08	4.91
**Sep**	0.14	5.10	3.90	1.77	2.86	0.12	2.72	2.28	1.77	4.57
**Oct**	0.04	6.27	6.62	1.88	3.39	0.15	3.87	2.13	1.88	5.31
**Nov**	0.07	2.00	3.00	1.72	4.02	0.05	3.55	2.00	3.01	6.26
**Dec**	0.02	9.54	4.00	1.75	2.98	0.25	6.43	2.57	2.35	5.50

Supplementary Table S5. The statistical details of the Long Ashton Weather Generator (LARS-WG5) validation results. Note that the LARS-WG5 does not perform the F-test for temperature.

Month	Rainfall						Maximum Temperature			
	KS	p-Value	t	p-Value	F	p-Value	KS	p-Value	t	p-Value
Jan	0.02	1.00	-0.55	0.59	1.14	0.73	0.05	1.00	-0.41	0.69
Feb	0.07	1.00	-0.79	0.43	1.21	0.61	0.05	1.00	-0.43	0.67
Mar	0.14	0.98	-0.02	0.98	1.53	0.26	0.11	1.00	0.63	0.53
Apr	0.09	1.00	0.80	0.43	1.18	0.66	0.11	1.00	-0.02	0.98
May	0.12	1.00	-0.93	0.36	1.02	0.95	0.16	0.91	0.97	0.34
Jun	0.44	0.02	0.33	0.74	1.10	0.80	0.05	1.00	-1.09	0.28
Jul	0.35	0.10	1.98	0.05	20.77	0.00	0.05	1.00	-0.83	0.41
Aug	0.44	0.02	-0.11	0.92	1.17	0.67	0.05	1.00	-0.34	0.74
Sep	0.26	0.36	-1.19	0.24	2.70	0.01	0.11	1.00	0.71	0.48
Oct	0.12	0.99	0.11	0.91	1.71	0.15	0.11	1.00	1.20	0.24
Nov	0.09	1.00	-0.19	0.85	1.10	0.80	0.05	1.00	1.23	0.22
Dec	0.18	0.83	0.25	0.81	1.71	0.16	0.11	1.00	0.60	0.55

Supplementary Table S6. Description of the five Global Circulation Models (GCMs) used

Developer	Developer Acronym	Country	Global Climate Model	Grid Resolution	
				Longitude	Latitude
Beijing Climate Center Climate System Model, China Meteorological Administration	BCC	China	BCC_CSM1.1(m)	1.13	1.13
Meteorogical Research Institute	MRI	Japan	MRI-CGCM3	1.00	0.50
Institut Pierre-Simon Laplace	IPSL	France	IPSL-CM5B-LR	3.75	1.89
Commonwealth Scientific and Industrial Research Organization/Queensland Climate Change Centre of Excellence	CSIRO-QCCCE	Australia	CSIRO-Mk3-6-0	1.88	1.88
National Oceanic and Atmospheric Administration, Geophysical Fluid Dynamics Laboratory	NOAA GDFL	USA	GFDL-ESM2M	2.50	2.00

Supplementary Table S7. List of parameters used to calibrate the Food and Agriculture Organization (FAO) AquaCrop model to Lilongwe District Conditions

Parameter	Lilongwe Calibration	FAO Calibration	Reference/Source
Daily climate data (rainfall, ET_o and temperature, wind speed, relative air humidity)	01 Oct 1999 to 30 Sep 2000	n/a	Chitedze Agricultural Research Station
CO_2 concentration	Mauna Loa Observatory	Mauna Loa Observatory	AquaCrop database
Type of planting	Direct sowing	n/a	Default in AquaCrop
Plant density	4.7 plants ha^{-1}	6.5-7.5 plants ha^{-1}	Literature[58]
Initial canopy cover	0.31%	0.49%	Automatically adjusted in AquaCrop
Maximum canopy cover	70%	96%	Adapted from literature[40]
Maximum effective rooting depth	0.6 m (shallow rooted)	2.3 m	Chitedze Agricultural Research Station; Literature[40]
Reference harvest index (HI)	38	48	Literature[60]
Effects of soil fertility stress	Considered	Not considered	Literature[17,59]
Relative biomass production	Very poor (35 %)	n/a	Adapted from literature[40]
Reduction in maximum canopy cover	64%	Not considered	Adapted from literature[40]
Reduction in canopy expansion	6%	Not considered	Adapted from literature[40]
Average decline in canopy cover	0.10 % day^{-1}	Not considered	Adapted from literature[40]
Reduction in water productivity	47%	Not considered	Adapted from literature[40]
Sowing date	Rainfall onset between 15 Nov and 31 Dec	n/a	Literature[61]
	Based on 30 mm in 5-day period (2nd occurrence)	Based on 80 mm cumulative rainfall	Literature[43]
Crop development (phenology)	Growing degree-days	n/a	Default in AquaCrop
Time to emergence	72	80	Chitedze Agricultural Research Station
Time to maximum canopy cover	768	705	Chitedze Agricultural Research Station; Literature[43]
Time to maximum rooting depth	1416	1409	Literature[43]
Start of canopy senescence	1200	1400	Literature[40,43]

Time to maturity	1548	1700	Chitedze Agricultural Research Station; Literature[40,43]
Time to flowering	792	880	Chitedze Agricultural Research Station; Literature[40, 43]
Length building up HI	749	750	Chitedze Agricultural Research Station; Literature[43]
Duration of flowering	108	180	Chitedze Agricultural Research Station; Literature[43]
Soil fertility	Poor (35%) i.e. 65% stress	Non-limiting (100%)	Literature[17,59]
Soil type	Deep uniform sandy clay loam	Deep loamy	Literature[33]
Soil thickness	2.10 m	4.00 m	Literature[40]
Permanent wilting point	14.9 vol %	20.0 vol %	Literature[40]
Field capacity	25.8 vol %	32.0 vol %	Literature[40]
Saturation point	44.1 vol %	47.0 vol %	Literature[40]
Total water available	109 mm m^{-1}	120 mm m^{-1}	Literature[40]
Saturated hydraulic conductivity	360 mm day^{-1}	125 mm day^{-1}	Literature[40]
